# Mid and long-term overall survival after carcinologic resections of thyroid cancer bone metastases

**DOI:** 10.3389/fsurg.2022.965951

**Published:** 2022-07-12

**Authors:** Henri Fragnaud, Jean-Camille Mattei, Louis-Romée Le Nail, Mỹ-Vân Nguyễn, Thomas Schubert, Anthony Griffin, Jay Wunder, David Biau, François Gouin, Paul Bonnevialle, Gualter Vaz, Mickael Ropars, Vincent Crenn

**Affiliations:** ^1^ Orthopedics and Trauma Department, University Hospital Hotel-Dieu, CHU Nantes, Nantes, France; ^2^ Ramsay Santé, Hôpital Privé Clairval, Marseille, France; ^3^ Département d’Orthopédie, Aix Marseille Université, APHM, Marseille Medical Genetics (MMG), Hôpital NORD, Marseille, France; ^4^ Orthopedics and Trauma Department, University Hospital, of Tours, Tours, France; ^5^ CNRS ERL 7001 LNOX: Leukemic Niche and Redox Metabolism – EA 7501 GICC (Groupe Innovation et Ciblage Cellulaire), Université de Tours, Tours, France; ^6^ Department of Orthopedic Surgery, Institut de Recherche Expérimentale et Clinique (IREC), Cliniques Universitaires Saint Luc, Université Catholique de Louvain (UCLouvain), Brussels, Belgium; ^7^ Division of Orthopaedic Surgery, Musculoskeletal Oncology Unit, Sinai Health System, Toronto, ON, Canada; ^8^ Orthopedic Department, Cochin Hospital, AP-HP, Paris, France; ^9^ Surgery Department, Léon Bérard Center, Lyon, France; ^10^ Orthopedic and traumatology surgery department, Riquet Pierre-Paul Hospital, Toulouse, France; ^11^ Department of Orthopedic Surgery, Pontchaillou University Hospital, Rennes, France; ^12^ CRCI2NA (Centre de Recherche en Cancérologie et Immunologie Nantes-Angers), INSERM UMR 1307, CNRS UMR 6075-Team 9 CHILD (Chromatin and Transcriptional Deregulation in Pediatric Bone Sarcoma), Nantes Université, Nantes, France

**Keywords:** bone metastasis, thyroid cancer, wide resection, long term, overall survival

## Abstract

**Background:**

Bone metastases in thyroid cancer impair the patient's quality of life and prognosis. Interestingly, wide margins resection as the surgical treatment of bone metastases might improve the overall survival (OS). Nonetheless, data are lacking regarding the potential benefits of this strategy.

**Methods:**

In order to assess the OS of patients with thyroid cancer after a bone metastases carcinologic resection, a retrospective multicentric study was performed, evaluating the 1, 5, 10 and 15 years-OS along with the potential prognosis associated factors.

**Results:**

40 patients have been included in this multicentric study, with a mean follow-up after surgery of 46.6 ± 58 months. We observed 25 (62.5%) unimestastatic patients and 15 multimetastatic patients (37.5%). The median overall survival after resection was 48 ± 57.3 months. OS at 1, 5, 10, and 15 years was respectively 76.2%, 63.6%, 63.6%, and 31.8%. Survival for patients with a single bone metastasis at 15 year was 82.3%, compared with 0.0% (Log Rank, *p* = 0.022) for multi-metastatic bone patients.

**Conclusions:**

This study advocates for an increased long term 10-year OS in patients with thyroid cancer, after resection of a single bone metastasis, suggesting the benefits of this strategy in this population.

## Introduction

Over the last three decades, the incidence of thyroid cancer has been on the rise (1). Despite an excellent associated overall survival rate (80%–95% at 10 years) (2–6), bone metastases in this disease can dramatically decrease the functional and overall prognosis. Indeed, bone metastases represent the second most frequent sites of systemic spread after lungs, ranging from 2 to 15% of all thyroid cancer types combined (7, 8). Leading to skeletal related events (SRE), bone metastases are associated with a significant decrease in quality of life, particularly regarding their functional impact, with pain and fracture risk (7, 8). In this context, looking for bone metastases is part of the systematic assessment not only during the oncologic work-up, but also following primary treatment (9). Besides, as medical treatment has been considerably improved these recent decades, giving better overall survival of patients with thyroid cancers, it does allow increasingly ambitious surgeries such as wide (R0) or marginal (R1) carcinologic resections in bone metastases management (6, 10–12). In this way, resection of a bone metastasis might be associated with better overall survival and could even be considered as curative (8, 11, 13, 14). Although wide resection could achieve better disease control, there are potential functional issues as they are associated with anaesthesia burdens, greater surgical sacrifices and potential complications (15–17). Hence, thyroid and skeleton tumour Multi Disciplinary Team (MDT) discussions attempt to identify which patient might benefit the most from these surgeries, so as to provide wide resections similar to primitive bone tumour management, while also avoiding overtreatment.

Prognosis factors data for overall survival in carcinologic bone resections appear to be paramount, but remain unclear because of the scarcity of published studies on this subject (15, 16). Therefore, this retrospective, multi-centric study aims to evaluate overall survival in patients with thyroid cancer who underwent a carcinologic resection of bone metastasis, as well as potential prognostic factors associated at the time of bone metastasis resection.

## Materials and methods

### Study design

This was a retrospective multicentre study involving nine tertiary care referral centers, including seven in France (Nantes, Rennes, Tours, Marseille, Lyon, Toulouse, Paris), one in Canada (Toronto), and one in Belgium (Brussells). All included patients had wide margin “en bloc” resection for a bone metastasis from thyroid cancer (operated on between 1992 and 2018). The resection indication has been discussed in an MDT meeting or as a concerted decision between surgeons and oncologists, depending on patient-specific criteria such as prognosis, age, localization, and the number of metastatic localizations.

### Study objective

The main objective of the study was to assess overall survival (OS) in patients who have had an “en bloc” resection of a bone metastasis in thyroid cancer. This data was retrospectively collected either by analysis of patients' medical records, or by telephone call to the attending physicians or the patients themselves.

The secondary objectives were to analyse the effect of clinical and epidemiological factors associated at the time of the bone metastasis resection: such as the number of bone metastasis (single vs. multiple), the association to a visceral metastasis, and the impact of resection margin quality (R0 > 2 mm of the tumor or <2 mm with natural barrier, R1 incomplete microscopic resection, R2 intralesional macroscopic resection). We also collected other data and analysed possible links with OS survival in our cohort: age, sex, histological subtype, synchronous (identification of bone metastasis at time of thyroid cancer diagnosis or during the first six months) or metachronous (diagnosis of bone metastasis after the first six months*)*, year of metastasis resection surgery, Radioactive Iodine (RAI) Therapy, associated local radiotherapy, metastatic lesion localization (pelvic localisation or others), lesion size, pathologic fracture. A multivariate analysis was also performed to assess various factors statistical association with overall survival.

### Ethics and statistical analysis

The study was conducted according to the guidelines of the Declaration of Helsinki, and due to the non-interventional nature of the study, no approval from an ethics committee was necessary at the time of the beginning of the study. The study was reported to the “Direction de la Recherche Clinique” (DRC) of the University Hospital of Nantes, France. The requisite processes were undertaken with the “Commission Nationale de l’Informatique et des Libertés” (CNIL).

Quantitative variables are expressed as means and range; qualitative variables are presented as total number of events and percentages. All survival analyses were carried out by Log-Rank method (mantelcox). The multivariate Cox model regression analysis (selecting variables with univariate *p* < 0.10) was used to identify factors associated survival probability. The variables initially selected were exited from the equation by the ascending stepwise method (conditional likelihood ratio). The significance threshold used for our study was <0.05. Data was collected using Microsoft® Excel, and the statistical analyses were performed using IBM® SPSS Statistics V25 software.

## Results

The mean age at the time of surgery in the study cohort was 61.2 years (29–78 years), the sex ratio was 1.1, (21 men, 19 women). The average follow-up was 46.6 months, (0–225 months). The mean survival after surgery was 60.0% (16 patients died in our series). Tumor were located in 30% cases on the pelvic ring (iliac bone) (12 cases), and in 70% cases (28 cases) in other localizations (16 femurs, eight humerus, two scapula, one rib, one clavicle) (Table 1).

**Table 1:** Study population data.

Characteristics data	Study cohort
Age thyroid surgery	53.1 (23 ; 77)
Age bone surgery	61.2 (29 ; 78)
Man	19 (47.5%)
Women	21 (52.5%)
Adjuvant therapy	
Radiotherapy	16 (40.0%)
Chemotherapy	10 (25.0%)
Radioactive Iodine (RAI) Therapy	19 (72.5%)
Metastasis Data	
Unimetastatic Bone	
Yes	25 (62.5%)
No	15 (37.5%)
Associated visceral metastase	
Yes	18 (45.0%)
No	22 (55.0%)
Margins resection	
R0	25 (62.5%)
R1	7 (17.5%)
R2	8 (20.0%)
Pathologic Fracture	
Yes	11 (27.5%)
No	29 (72.5%)
Bone Metastasis Location	
Pelvic ring	12 (30.0%)
Others	28 (70.0%)
Histological subtype	
Papillary	25 (62.5%)
Vesicular	2 (5.0%)
Medullary	2 (5.0%)
Unknown	11 (27.5%)

### Global overall survival

**Figure 1:**
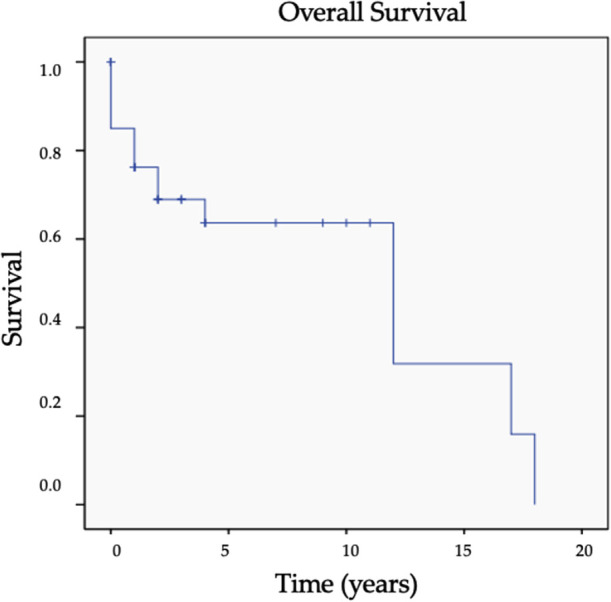
Overall survival curve (cross represents the censored data).

The median overall survival after bone metastasis resection was 48 ± 57.3 months. Overall survival at 1, 5, 10, and 15 years follow-up were 76.2%, 63.6%, 63.6%, and 31.8% (Figure 1).

### Unimetastatic vs. multimetastatic

**Figure 2:**
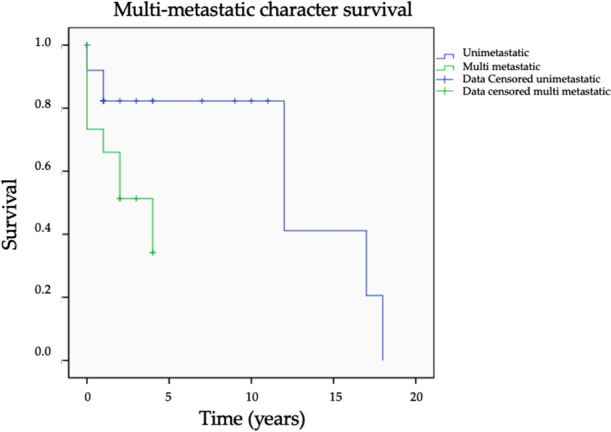
Survival curve for single bone metastasis and plurimetastatic patients (*p* = 0.022 Log Rank).

Survival for patients with a single bone metastasis at 1, 5, 10, years was respectively 82.3%, 82.3%, 82.3% compared with 66.0%, 0.0%, 0.0% (Log Rank, *p* = 0.022) for patients with multiple bone metastases (Figure 2, Table 2).

**Table 2:** Univariate analysis of outcomes for the whole series.

Characteristics	Number of patients	1-year overall survival (%)	5-year overall survival (%)	10-year overall survival (%)	*p* value by the log-rank test
Bone metastasis					
Unimetastatic	25 (62.5%)	82.3%	82.3%	82.3%	**.** **022**
Multimetastatic	15 (37.5%)	66.0%	34.2%	–	
Association with visceral metastasis					
Yes	18 (45.0%)	55.6%	38.9%	38.9%	**.** **028**
No	22 (55.0%)	94.1%	86.9%	86.9%	
Margins					
R0	25 (62.5%)	86.9%	68.7%	68.7%	
R1	7 (17.5%)	85.7%	71.4%	71.4%	.058
R2	8 (20.0%)	50.0%	37.5%	37.5%	

*The bold value are significant.*

### Association with visceral metastasis

Survival for patients with associated visceral metastasis at 1, 5, 10, years was respectively 55.6%, 38.9%, 38.9% compared with 94.1%, 86.9%, 86.9% (Log Rank, *p* = 0.022) for those with bone metastasis only. Due to crossing curves the Log-Rank test result has to be used with caution (*p* = 0.028) (Supplementary Figure S1, Table 2).

### Margins analysis

Survival for patients with R0 resections at 1, 5, 10, years was respectively 85.7%, 68.7%, 68.7% compared with 85.7%, 71.4%, 71.4% for R1 and 50.0%, 37.5%, 37.5% for R2 resections. Due to crossing curves and small cohort, the Log-Rank test result has to be analysed with caution (*p* = 0.058) (Supplementary Figure S2, Table 2).

### Other factors

The OS for patient who had synchronous (at the diagnosis) metastasis was at 1, 5, 10 years 91.7%, 80.2%, 80.2%, and 66.2%, 55.1%, 55.1% for patient who had metachronous (over 6 months) metastasis, yet the difference was not significant (Log-Rank *p* = 0.220) (Table 3).

**Table 3:** Univariate log-rank analysis of the overall survival (OS) for the whole series.

Characteristics	Number of patients	1-year OS (%)	5-year OS (%)	10-year OS (%)	*p-*value by the log-rank test
Delay of diagnosis					
Metachronous	24 (60.0%)	66.2%	55.1%	55.1%	.220
Synchronous	16 (40.0%)	91.7%	80.2%	80.2%	
Pathologic fracture					
Yes	29 (72.5%)	78.0%	60.6%	–	.480
No	11 (27.5%)	72.7%	66.1%	66.1%	
RAI Therapy					
Yes	29 (72.5%)	85.4%	74.7%	74.7%	.045
No	11 (27.5%)	54.5%	27.3%	–	
Radiotherapy					
Yes	24 (60.0%)	56.3%	49.2%	49.2%	.021
No	16 (40.0%)	90.5%	74.3%	74.3%	
Tumor size					
<100 mm	33 (82.5%)	81.5%	70.7%	70.7%	.093
>100 mm	7 (17.5%)	42.9%	21.4%	21.4%	
Location					
Pelvic ring	12 (30.0%)	91.7%	59.4%	59.4%	.542
Other locations	28 (70.0%)	78.0%	65.6%	65.6%	
Histological Type					
Unknown	11 (27.5%)	72.7%	72.7%	72.7%	
Medullary	2 (5.0%)	–	–	–	.440
Papillary	25 (62.5%)	74.7%	63.4%	63.4%	
Vesicular	2 (5.0%)	–	–	–	

*RAI, Radioactive Iodine therapy.*

The OS for patients without fracture was 72.7%, 66.1%, 66.1% at 1, 5, 10 years respectively, compared to 78.0%, 60.6% and 0.0% for patients who had a pathological fracture. This criterion does not seem to have any influence in the short term, however in the long term the fracture seems to be pejorative, but it did not reach significance (Log-Rank *p* = 0.480).

Association with RAI therapy seemed to improve OS at 1, 5, 10 and 15 years: 85.4%, 74.7%, 74.7%, 37.4% compared to 54.5%, 27.3%, 0.0%, 0.0% (Log-Rank *p* = 0.045). Local radiation therapy (RT) was associated to lower OS at 10 years (49.2% vs. 74.3%) (Log-Rank *p* = 0.021).

The OS for patients with tumour size <100 mm was 81.5%, 70.7%, 70.7%, at 1, 5, and 10 years respectively, compared to 42.9%, 21.4%, and 21.4% for patients with a lesion >100 mm. Size <100 mm seems to show improved OS without reaching significance (Log-Rank *p* = 0.093). Location did not seem to have an impact on overall survival. The OS at 1, 5, 10 years was 91.7%, 59.4%, 59.4%, for the pelvic location, 78.0%, 65.6%, 65.6% for other locations (Log-Rank, *p* = 0.542).

### Multivariate analysis

Each variable with an univariate log-rank analysis significance threshold of *p* < 0.10 was added to the multivariate analysis model: plurimetastatic presentation (*p* = 0.022), resection margins (*p* = 0.058), associated visceral metastasis (*p* = 0.028), RAI therapy (*p* = 0.045), associated radiation therapy (*p* = 0.021), Tumor size >100 mm (*p* = 0.092). Variables initially selected and exited from the equation by the ascending stepwise method (conditional likelihood ratio): resection margins (score: 3.15; degree of freedom (df): 2; *p* = 0.207), associated visceral metastasis (score: 3.15; df: 1; *p* = 0.112), RAI therapy (score: 2.68; df: 1; *p* = 0.102), associated radiation therapy (score: 2.85; df: 1; *p* = 0.091). HR: Hazard Ratio. Model *p*-value = 0.017.

Multivariable Cox Proportional Hazards model analysis of survival was performed utilizing various patient and tumour factors. It involved initially all univariate Log-Rank analysis with *p* < 0.10, comprising the plurimetastatic presentation (*p* = 0.022), associated visceral metastasis (*p* = 0.028), resection margins (*p* = 0.058), RAI therapy (*p* = 0.045), associated Radiation therapy (*p* = 0.021), Tumor size >100 mm (*p* = 0.092).

In this analysis, using an ascending stepwise method (conditional likelihood ratio), the only two variables remaining in the final model were the plurimetastatic presentation (*p* = 0.022), and tumor size >100 mm (*p* = 0.056) (Table 4). The multivariate Cox regression model revealed a 79.3% overall predictive value.

**Table 4:** Multivariate Cox regression model for the overall survival probability.

Multivariate analysis	HR (95.0% CI)	*p*-value
Plurimetastatic presentation	4.21 (1.23–14.45)	*p* = 0.022
Tumor size >100 mm	3.06 (0.97–9.64)	*p* = 0.056

## Discussion

Bone metastases management has become a major issue in cancer, particularly in thyroid cancer. Moreover, it has been established that bone metastases impair survival of patients and their quality of life (18). Oncological wide resection of bone metastasis is more and more performed as the prognosis of these cancers improves (due to medical progresses such as targeted therapies or immunotherapy). Furthermore, it seems important to keep in mind the advantages of resection and reconstruction over preventive fixation in bone metastasis: implant stability, reduced risk of local disease progression, survival improvements, and reduced risk of implant failure in patients are most often highlighted (15, 19–21). However, its causality effect on survival is still debatable (15, 16), and the draw-backs of this strategy are the higher surgical and anesthesiologic risks, with notables infection rates, dislocation complications in the case of hip arthroplasty and potential anatomical structure sacrifices in cases of a wide resection.

Currently, such wide resection management remains a collegial decision, to be considered on a case-by-case basis in a multidisciplinary setting (20–22), in order to judge the benefit/risk ratio in these rare surgeries. Wide resection is frequently performed in a single bone metastasis setting. Since bone metastasis surgical management shoul become more and more frequent, it is of great interest to evaluate OS in resection cases, in order to identify which patients might benefit from this attitude. It will help decision-making for both clinicians and patients.

### Overall survival

The wide resection strategy appears to achieve high OS for the patients with thyroid cancer involving bone. It can be noted that the survival after resection in our study is high; indeed, we observed an OS of 76.2%, 63.6%, 63.6% and 31.8% at 1, 5, 10 and 15 years respectively. Our study, focusing on the surgical scope with a 40 patients cohort demonstrates excellent OS results when compared to the current literature (7, 13, 15, 19, 20, 23). Two other cohorts focused on this topic in thyroid cancer. The one by Satcher et al. in 2012 (15), with 41 patients over 23 years of data collection (1988 to 2011), 80.0% of patients had a carcinological resection of bone metastasis. Their survival rate was 72.0% at 1 year, 29.0% at 5 years and 20.0% at 8 years. Nakayama et al. reported slightly better results in 2014 (16), with 40 patients over 14 years (1994 to 2008), 67.0% of patients having wide resection for oligometastatic disease, with overall survival at 2, 5 and 10 years of 77.2%, 64.3% and 45.7%, respectively.

Our work demonstrates higher OS than these two cohorts. This difference can mainly be explained by the selection criteria differences, as we excluded palliative intralesional surgery solutions which are offered to patients with more advanced disease and therefore of poorer prognosis. Secondly, it might also be explained as their series included more bony multi-metastatic patients with 70.0% for Satcher et al, 52.5% for Nakayama et al and 37.5% in our series (15, 16). Moreover, we must also take into account the improvement of oncological treatments on survival since our study was carried out almost ten years after the two others mentioned above (1). It should also be noted that there is heterogeneity in our series since the treatments evolved over the series time period between the 1990s, 2000 and 2010, which allows us to smooth out this difference.

In view of the high survival rates obtained with more than half patients alive at 10-years follow-up, it is therefore necessary to look for specific criteria to allow a better selection of candidates for this surgery.

### Factors influencing overall survival

#### Isolated metastasis

We observed a significant improvement in survival when metastatic resection surgery was performed in patients with a single bone metastasis, OS at 10 years of 82.3% versus 0.0% (Log-Rank *p* = 0.022) in multi-metastatic patients. This result is in agreement with the literature, notably with Satcher et al. who showed a clear trend without significance, and Nakayama et al. who showed significantly higher survival in this population compared to bone polymetastatic patients (15, 16). The unimetastatic patients in our cohort had the same survival rate at one (82.3%) and ten years (82.3%), suggesting a potential remission effect.

Moreover, it must be noted that a multimetastatic presentation was the only significant variable in our multivariate Cox Model regression with a Hazard Ratio of 4.21 (1.23–14.45), (*p* = 0.022). It is therefore the most critical factor to consider before proposing a carcinological resection strategy in thyroid bone metastasis. Multimetastatic patients with an apparent worse prognosis than unimetastatic patients should be regarded with caution before proposing a functionally impairing surgical procedure such as pelvic or axial bone resection.

#### Association with visceral metastases

We highlight a clear trend without significance on this criterion, as OS at 10 years was 86.9% in no visceral metastasis cases vs. 38.9% in patients with associated visceral metastases. The data in the literature remains in agreement with our results for Nakayama et al., their results are in line with ours since the association with visceral metastases reflects a more advanced disease and therefore of a poorer prognosis (16).

#### Margins

In our study, R0 or R1 margins seemed to improve prognosis compared to R2, with a median survival of 17 years for R0, 12 years for R1 and 1 year for R2; and with 68.7% and 71.4% 10-years OS for R0 and R1 surgeries, compared to R2 surgeries (37.5%).

We found that it may be beneficial to perform extra-lesional surgery on metastatic thyroid bone metastasis when feasible, but it remains unclear if it is mandatory to perform a wide resection in this context, as R1 margins had similar 10-year OS as R0 margins, furthermore with the presence of a pathological fracture. It should also be noted that R2 resections probably reflect more advanced or too advanced disease to be treated with extra-lesional resection. Finally, it shows that there might be a benefit in performing a macroscopic marginal excision, as survival is greatly reduced for patients who have had a R2 resection in our cohort, but there might be some confusing bias as we did not identify margin quality as a significant factor in our multivariate analysis. Nonetheless, this criterion is all the more interesting as it is scarcely mentioned in the literature and could be a decisive factor by analogy with primary bone tumors (24) or metastases of other cancers, such as resection of a solitary metastasis in renal cell carcinoma or other malignancies (12, 25, 26). There is indeed a difference in survival depending on the quality of the macroscopically resection, but our small cohort might lack power on this point to highlight significance.

#### Others data

With regard to adjuvant therapies, our study found that the combination with RAI therapy was associated with higher survival of 74.7% at 5 years compared 27.3% without RAI therapy (Log-Rank *p* = 0.045), which are in line with previous studies, as RAI-sensitive thyroid cancer subtypes are known to be of better prognosis (8). Adjuvant radiotherapy seems to be associated with lower survival of 49.2% compared to 74.3% (Log-Rank *p* = 0.021). It might be the result of a selection bias, as RAI-refractory patients will mostly be treated with associated radiotherapy, as well as patients with more advanced disease or intraleseionnal surgeries. RAI is known as a protective therapy in thyroid cancers after total thyroidectomy (15, 27). Developing a bone metastasis while having RAI therapy would therefore mean a more advanced or aggressive disease. However, our results, demonstrated a survival improvement for patient who did benefit from RAI therapy in combination with a surgery of their bone metastasis. Adjuvant RT and RAI therapy were nevertheless not identified in our multivariate Cox model as significant variables influencing survival. In contrast, tumor lesion size >100 mm almost reached significance with a HR = 3.06 (0.97–9.64), (*p* = 0.056). Massive lesions seemed to be associated to a lower OS in our cohort.

### Limitations, bias and strengths

The limitations of our series are primarily the fact that data were collected retrospectively. However, given the small number of patients there are no alternatives on this rare topic. Furthermore, some data are still poorly informed such as the histological type. This is a multicentric, and heterogeneous series. We did not take into account relapse or local recurrence free survival and only focused on OS. We must keep in mind the progresses in the oncological medical management of thyroid cancer that also improve OS; in this we omit the endocrine oncological management, which also plays a role in OS; these medical progresses might also, in the future, modify our bone metastasis resection indications. We also disregard the management of other (bone or visceral) metastasis in addition to the operated one, nor the ECOG performance status. Lastly, it should be noted that we could not deduce causality effects due to our study design and relatively low numbers, and we only observed statistical associations, and some confusion bias might be present.

The strength of our study is that it is an original work with a relatively consequent cohort on this rare subject, in order to get a picture of OS with a long follow up (up to 15 years). We had a surgical focus on simple clinic and epidemiologic factors, using a complementary univariate and multivariable approach. It allowed us to refine the indications of these surgeries which remains little studied in the last years, and it might help clinicians and multidisciplinary teams to decide on a case-by-case basis.

### Conclusions

Our study shows that the wide resection of a single bone metastasis in the context of thyroid cancer seems to be associated with higher survival rates, and might be an appropriate option to be considered in specific conditions. This decision must remain the result of a multidisciplinary discussion between orthopaedic surgeons and oncologists. Moreover, we identified those patients who had single bone metastasis without visceral involvement might have a higher OS, and that most of them will live more than 10 years after bone metastasis surgical resection.

## Data Availability

'The original contributions presented in the study are included in the article/Supplementary Materials, further inquiries can be directed to the corresponding author/s.
