# Supplementary figures and images for: Mid and long-term overall survival after carcinologic resections of thyroid cancer bone metastases
Source: Front Surg. 2022 Jul 12;9:965951. doi: 10.3389/fsurg.2022.965951 (PMC9314764; doi:10.3389/fsurg.2022.965951)

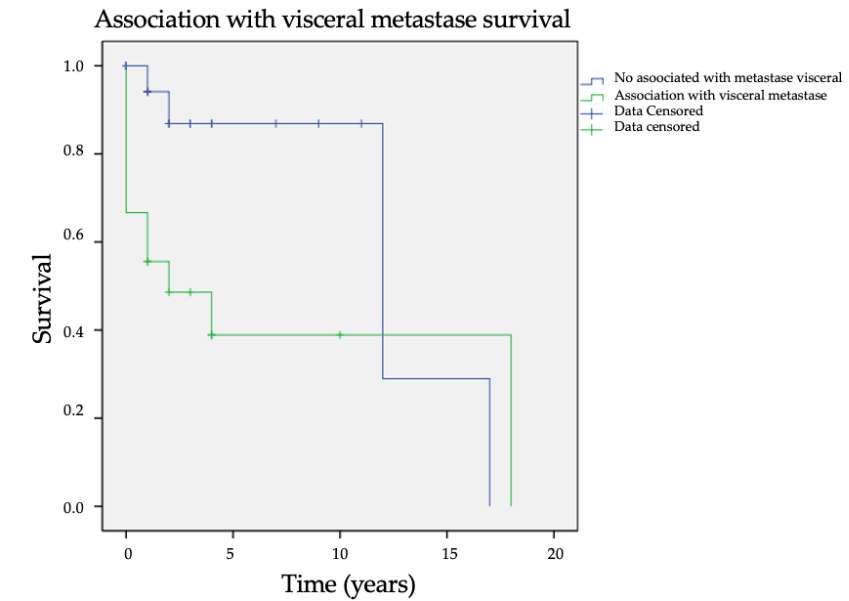

Supplement: Supplementary file 1 [file Image_1_v1.tif]

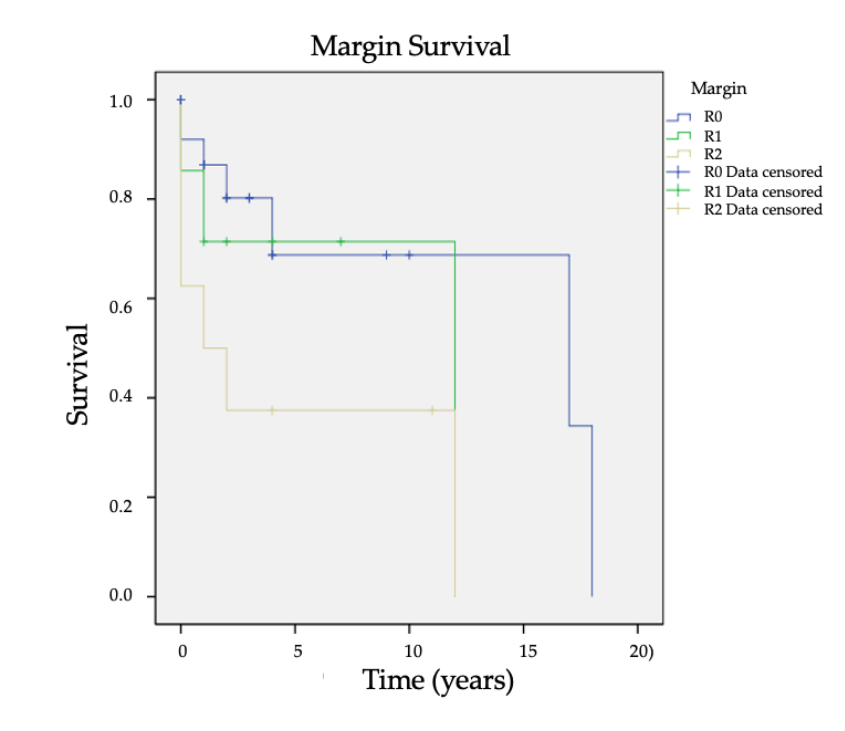

Supplement: Supplementary file 2 [file Image_2_v1.tif]
